# Skull-Base Plasmacytomas: A Systematic Review on Therapeutic Trends

**DOI:** 10.3390/cancers18152495

**Published:** 2026-08-04

**Authors:** Francisco Call-Orellana, Kishore Balasubramanian, Hanyu Qiu, Alexander Houpt, Tushar Kanti Bhadra, Georgios Toumbas, Beste Gülsuna, Jeffrey Zuccato, Panayiotis Pelargos, Abdul Basit Khan, Hakeem J. Shakir

**Affiliations:** 1 Department of Neurosurgery, MD Anderson Cancer Center, The University of Texas, Houston, TX 77030, USA; facall@mdanderson.org; 2 College of Medicine, Texas A&M Health Science Center, Houston, TX 77807, USA; kishore.b@tamu.edu; 3 Department of Neurosurgery, University of Oklahoma College of Medicine, Oklahoma City, OK 73104, USA; hanyu-qiu@ou.edu (H.Q.); alexander-houpt@ou.edu (A.H.); beste-gulsuna@ou.edu (B.G.); jeffrey-zuccato@ou.edu (J.Z.); panayiotis-pelargos@ou.edu (P.P.); abdul-khan@ou.edu (A.B.K.); 4 Department of Cardiovascular and Thoracic Surgery, National Institute of Cardiovascular Diseases, Dhaka 1207, Bangladesh; drtushar106@gmail.com; 5 Clinical School of Medicine, University of Cambridge, Cambridge CB2 0SP, UK; gt416@cam.ac.uk

**Keywords:** plasmacytoma, skull base, systematic review

## Abstract

**Background:** Skull base plasmacytomas are rare plasma cell neoplasms arising from the clivus, sphenoid bone, and petrous apex, presenting with cranial neuropathies and bearing a high risk of progression to multiple myeloma. Optimal management remains poorly defined due to their rarity. **Objective:** The objectives of this study are to comprehensively characterize the clinical presentation, management strategies, and outcomes of skull base plasmacytomas and identify evolving trends in the literature. **Methods:** A systematic search of PubMed, EMBASE, Web of Science, and Cochrane databases was conducted, yielding 92 studies (18 case series and 74 case reports) encompassing 118 patients. Data on the demographics, symptoms, imaging, histopathology, treatment, and outcomes were extracted and analyzed. **Results:** The median patient age was 56 years; visual disturbances (66.1%) and headache (54.2%) were the most common presenting symptoms. The middle and posterior cranial fossae were most frequently involved, and cranial nerve VI was most affected (65.7%). Surgical resection was performed in 63.6% of cases with a gross total resection in 36%. At a median follow-up of 12 months (calculated across the entire cohort of 118 patients, including the 29 who presented with a prior diagnosis of multiple myeloma), 81.4% were alive, with symptom resolution at 38.1% or improvement at 26.2%. Recurrence occurred in 19.4% of patients, and multiple myeloma developed in 25.8% of patients without a prior diagnosis. Multiple myeloma status was the only independent predictor of survival on multivariate analysis (OR = 10.14, 95% CI: 1.63–63.04, *p* = 0.013). **Conclusions:** Skull base plasmacytomas show consistent clinical patterns but variable management strategies. Surgery is increasingly utilized; yet, its survival benefit remains unclear. Prospective multicenter studies with standardized outcome reporting and molecular profiling are needed to optimize individualized care.

## Simple Summary

Skull base plasmacytomas are rare tumors made up of abnormal plasma cells that grow at the base of the skull, most commonly in the clivus, sphenoid bone, and petrous apex. Because so few cases exist, little is known about the best way to diagnose and treat them. In this systematic review, we pooled data from 92 published studies encompassing 118 patients to provide the largest pooled analysis of this tumor to date. We found that most patients presented with visual disturbances and headaches, and that the local invasion of the surrounding structures was extremely common. While surgery has been increasingly used in recent years, it did not improve survival compared to radiation-based treatment. The most important predictor of survival was whether patients developed multiple myeloma during follow-up. These findings highlight the need for long-term surveillance and future prospective studies to guide individualized treatment decisions.

## 1. Introduction

Plasmacytomas of the skull base are rare manifestations of plasma cell neoplasms, representing a small fraction of all head and neck tumors and an even smaller subset among plasma cell dyscrasias [1,2]. These tumors can present either as solitary lesions, termed solitary bone plasmacytoma (SBP) or extramedullary plasmacytoma (EMP) when in soft tissues, or as secondary lesions in the context of systemic multiple myeloma (MM) [1,3]. Epidemiologically, skull base plasmacytomas are most commonly diagnosed in middle-aged to older adults, with a mean age around 54 years, though cases in younger patients have been documented and are considered unusual [4,5]. The majority of skull base plasmacytomas originate from middle cranial fossa structures such as the clivus, sphenoid bone, and petrous apex, while a lesser subset of lesions occasionally arise from bones of the face [2,3].

**Figure 1:**
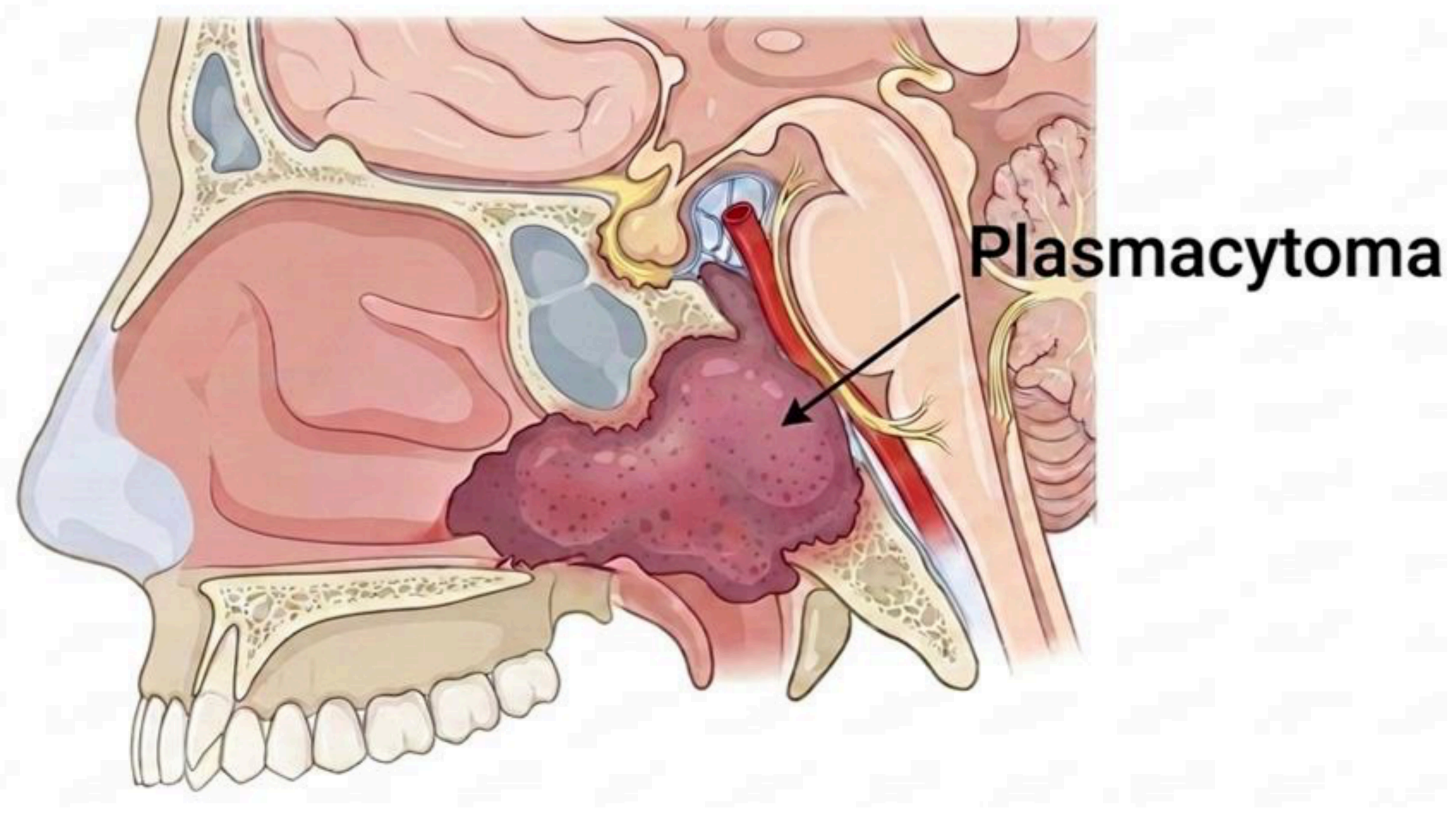
Representative illustration of a plasmacytoma of the skull base. This schematic illustration was drafted using the AI-assisted design tool FigureLabs (https://www.figurelabs.ai/, accessed 20 July 2026). The initial layouts were vectorized and subsequently manually refined, labeled, and verified by the authors to ensure scientific accuracy.

Pathophysiologically, plasmacytomas are characterized by the monoclonal proliferation of plasma cells, which secrete immunoglobulins and may infiltrate bone or soft tissue [1]. The distinction between solitary plasmacytoma and multiple myeloma is critical. According to the International Myeloma Working Group (IMWG) criteria, a solitary plasmacytoma requires a biopsy-proven single clonal lesion of bone or soft tissue, a normal skeletal survey and axial MRI (or CT) of the spine and pelvis apart from the primary lesion, and the absence of end-organ damage attributable to a clonal plasma-cell disorder. A variant termed solitary plasmacytoma with minimal marrow involvement permits clonal marrow plasma cells below 10%. By contrast, multiple myeloma is defined by clonal bone marrow plasma cells of at least 10% (or a biopsy-proven plasmacytoma) plus one or more myeloma-defining events, including CRAB features, or biomarkers of malignancy [6]. Skull base plasmacytomas, particularly those arising from the clivus, sphenoid, or petrous apex, carry a high risk of progression to multiple myeloma, with reported rates of conversion exceeding 50% within two years of diagnosis (Figure 1) [1,3,7]. Genetic alterations, including deletions in *p53*, 13q14, and translocations involving immunoglobulin heavy chain loci, have been implicated in disease progression, though the molecular mechanisms remain incompletely understood [8].

Clinical presentation is typically dominated by symptoms related to the mass effect (such as headache, visual disturbances, and vomiting) and cranial nerve involvement, with the abducens nerve most frequently affected [1,3]. Diagnostic evaluation involves imaging, particularly MRI and CT, followed by a histopathologic confirmation with immunohistochemical staining for plasma cell markers and light chain restriction [3]. A comprehensive systemic workup is essential to rule out underlying or concurrent multiple myeloma [1,3].

Given the rarity of skull base plasmacytomas, the limited number of reported cases, and their high risk of progression to multiple myeloma, a comprehensive synthesis of the existing literature is essential to better understand epidemiology, pathophysiology, clinical presentation, and management. This systematic review aims to report evidence-based practices, improve the diagnostic accuracy, and optimize the outcomes for affected patients, while also providing an update on the existing literature and highlighting gaps that warrant further research [2].

## 2. Materials and Methods

### 2.1. Literature Search

A systematic review was conducted using the Preferred Reporting Items for Systematic Reviews and Meta-Analyses (PRISMA) guidelines (Supplementary Files S1 and S2). The review was not prospectively registered in PROSPERO or another protocol registry, which we acknowledge as a limitation. PubMed, EMBASE, Web of Science, and Cochrane were searched from database inception to February 2025, operating the Boolean full-text search [(“plasmacytoma” OR “extramedullary plasmacytoma” OR “solitary bone plasmacytoma”) AND (“skull base” OR “clivus” OR “sphenoid” OR “petrous apex”)]. The same controlled-vocabulary and free-text terms were applied to each database, adapting the syntax to each platform: in PubMed, the strategy combined MeSH terms (“Plasmacytoma”[Mesh] and “Skull Base”[Mesh]) with the title/abstract keywords above; EMBASE used the corresponding Emtree terms with .tw/.mp field tags; and Web of Science and Cochrane (CENTRAL) used the same keyword strings within Topic/Title–Abstract–Keyword fields. No date, language, or document-type filters were applied at the database level; eligibility restrictions were applied only at screening. Reference lists of included studies and of relevant prior reviews were hand-searched, but a formal grey-literature search (e.g., trial registries, theses, and preprints) was not performed. Conference abstracts and proceedings were excluded a priori because they typically lack the individual patient-level clinical, treatment, and follow-up detail required for extraction in this analysis and are frequently superseded by subsequent full-text publications, raising the risk of duplicate counting. Studies were exported to Rayyan and duplicates were removed. To further mitigate the risk of duplicate inclusion of the same patient across overlapping case reports and case series, records were cross-checked for matching author groups, institutions, patient age and sex, tumor location, and treatment timeline, and apparent duplicates were resolved by retaining the most complete report.

### 2.2. Study Selection

Inclusion and exclusion criteria were defined as follows. Articles were included if they fulfilled the following criteria: (1) they report adult patients with histologically confirmed diagnosis of plasmacytoma and radiologically confirmed skull base location; (2) they include individual patient data including details of clinical factors, treatment, outcome, and follow-up; and (3) they were written in English. Studies were excluded if they fulfilled the. following criteria: (1) they were autopsy reports, animal studies, or studies focusing on imaging characteristics, genetics, or histopathology only; (2) they were conference abstracts, literature reviews, meta-analyses, systematic reviews, perspectives, or editorials; (3) they lacked adequate clinical data; (4) they were non-English or non-peer-reviewed sources; and (5) they were reports in the pediatric population, as plasmacytomas in this population differ meaningfully in biological behavior, treatment protocols, and diagnostic considerations, which would introduce clinical heterogeneity into the analysis.

Two independent reviewers (G.T. and T.K.B.) screened all titles and abstracts from the initial systematic search and assessed the full texts of articles that met the inclusion criteria. Two different reviewers (F.C.O. and K.B.) settled disagreements. Eligible papers were included. References of included studies were also screened to identify additional pertinent studies.

### 2.3. Data Extraction

Two reviewers (G.T. and T.K.B.) extracted data from each article, which was confirmed independently by two additional reviewers (F.C.O. and K.B.). Extracted data included authors, year published, sample size, age, gender, presenting symptoms and their duration, comorbidities, physical exam findings, radiological findings, surgical intervention and extent of resection, histopathological findings, adjuvant therapy received, time to recurrence, time of last follow-up with clinical status, and survival.

### 2.4. Data Analysis and Quality Assessment

The primary variables of interest were clinical characteristics, management strategies used, and treatment outcomes for patients with SBPs. For each study, two independent authors (F.C.O and T.K.B.) assessed the level of evidence using the 2011 Oxford Centre For Evidence-Based Medicine guidelines and the risk of bias by applying the Joanna Briggs Institute checklists for case reports and case series [9,10,11]. Meta-analysis was precluded because most included studies had levels IV–V of evidence (except for one level III with exhaustive individual patient data) and hazard ratios could not be deduced.

### 2.5. Statistical Analysis

Microsoft Excel (Microsoft Corporation, Redmond, WA, USA) was used for all data collection and analysis for central tendency and distribution. Continuous variables are summarized as medians with ranges and categorical variables are summarized as frequencies with percentages.

Pooled individual patient data allowed for univariate analysis using Chi-square or Fisher’s exact test and log-rank analysis plotted as Kaplan–Meier survival curves. Multivariate analysis was done using binary logistic regression. Statistical analysis was done using RStudio v2024.04.2. The multivariable model was specified as binary logistic regression predicting vital status (alive vs. deceased) at last reported follow-up rather than a Cox model, as individual-level time-to-event data were unavailable across the heterogeneous source studies. Given the limited number of outcome events relative to candidate predictors, the conventional events-per-variable threshold of ~10 was not met, and estimates should be interpreted as exploratory and hypothesis-generating rather than confirmatory. Categorical predictors in which all events fell within a single subgroup produced complete separation and are reported as non-estimable in the results.

## 3. Results

### 3.1. Study Inclusion

**Figure 2:**
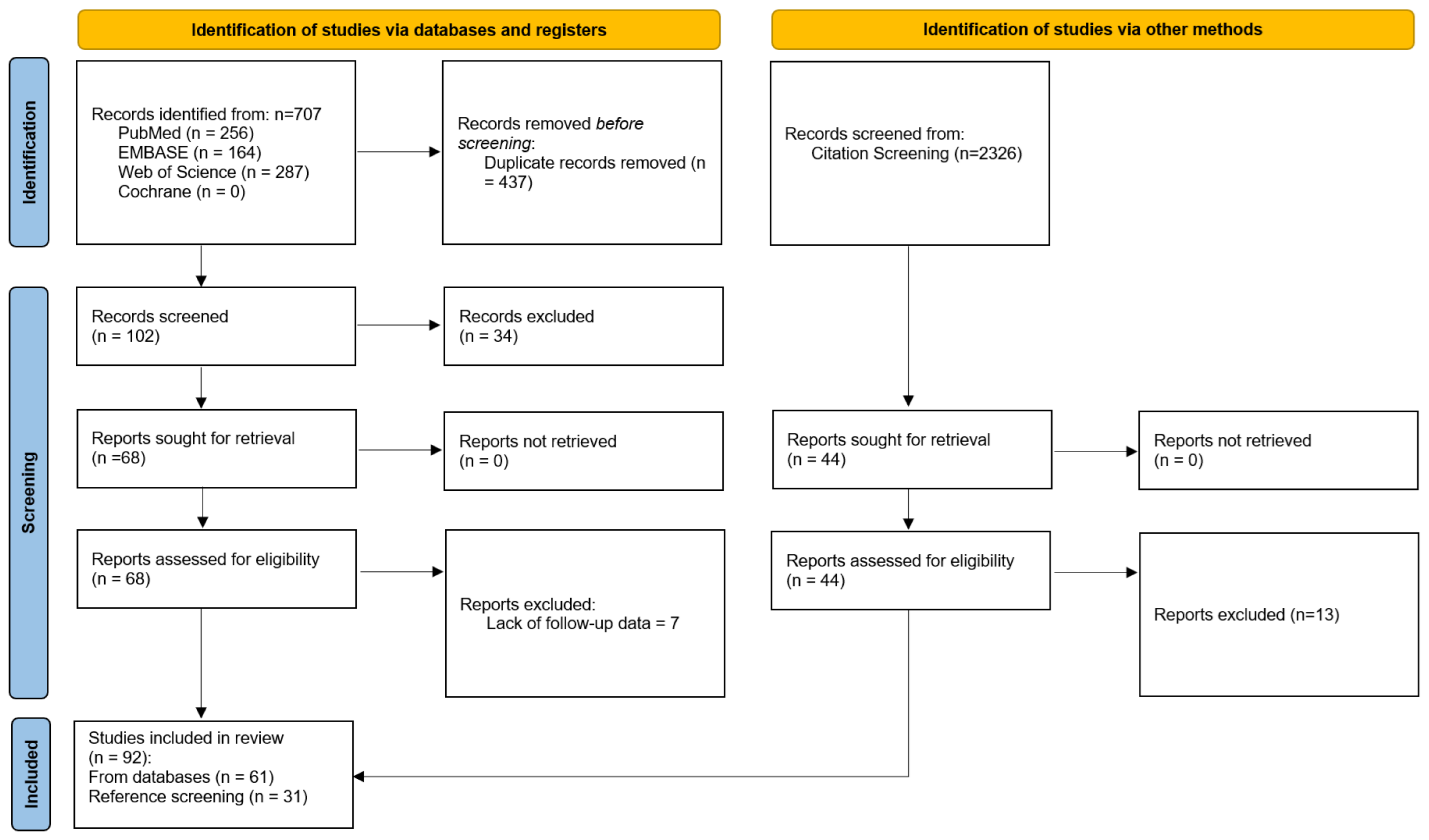
PRISMA flow diagram of the literature screening.

Figure 2 illustrates the flow diagram of the literature search. The search strategy yielded 707 studies (PubMed: 256, EMBASE: 164, Web of Science: 287, Cochrane: 0), of which 92 were included upon the pre-specified study inclusion criteria: 18 were case series [12,13,14,15,16,17,18,19,20,21,22,23,24,25,26,27,28,29] and 74 were case reports [1,3,30,31,32,33,34,35,36,37,38,39,40,41,42,43,44,45,46,47,48,49,50,51,52,53,54,55,56,57,58,59,60,61,62,63,64,65,66,67,68,69,70,71,72,73,74,75,76,77,78,79,80,81,82,83,84,85,86,87,88,89,90,91,92,93,94,95,96,97,98,99,100,101], with IV, and V levels of evidence, respectively (Table 1).

### 3.2. Patient Demographics and Clinical Presentation

A total of 118 patients with skull base plasmacytomas were included. The median age was 56 years (range, 19–81 years), and 65 patients (55%) were male. Among 85 patients with available data, the median duration of symptoms prior to diagnosis was 2 months (range, 0.07–66 months). A personal history of multiple myeloma was present in 29 patients (24.6%) out of 118 with available data, while 89 (75.4%) had no such history (Table 2).

The most common presenting symptoms were visual disturbances, reported in 78 patients (66.1%), and headache in 64 (54.2%), followed by motor disturbances in 18 (15.3%), and palpable mass or swelling in 15 (12.7%). Auditory disturbances (tinnitus, and hypoacusis) and dizziness/vertigo each occurred in 13 patients (11%). Sensory disturbances and ataxia/gait disturbance were each seen in 10 patients (8.5%). Less frequent symptoms included mechanical pain (*n* = 8, 6.8%), cognitive dysfunction (*n* = 7, 5.9%), dysphagia (*n* = 6, 5.1%), nausea/vomiting (*n* = 5, 4.2%), nasal discomfort (*n* = 5, 4.2%), speech problems (*n* = 3, 2.5%), and several non-specific symptoms each reported in a single case (0.8%).

Cranial nerve involvement was documented in 67 patients. The abducens nerve was most commonly affected (*n* = 44, 65.7%), followed by the trigeminal (*n* = 19, 28.4%), oculomotor (*n* = 11, 16.4%), facial (*n* = 7, 10.4%), optic (*n* = 6, 9%), trochlear (*n* = 6, 9%), vestibulocochlear (*n* = 4, 6%), hypoglossal (*n* = 3, 4.5%), and vagus (*n* = 1, 1.5%) nerves.

### 3.3. Clinical Workup

Initial imaging modality was reported for 113 patients. MRI was the most frequently used (*n* = 92, 81.4%), with lesions typically showing hypointense (*n* = 12, 10.6%) or isointense (*n* = 31, 27.4%) on T1-weighted imaging and isointense (*n* = 15, 13.2%) or hyperintense (*n* = 16, 14.2%) on T2. MRI-based contrast enhancement was reported for 67 patients with most having a homogeneous enhancement (*n* = 50, 42.4%). CT was performed in 69 patients (61.1%), X-ray in 19 (16.8%), PET scan in 9 (7.9%), and MRA in 3 (2.6%) (Table 3).

The tumor location was reported for all 118 patients. The middle cranial fossa was the most common site (*n* = 57, 48.3%), followed by the posterior fossa (*n* = 40, 33.9%), a tumor involving both the middle and posterior fossa (*n* = 9, 7.6%), a non-specified skull base location (*n* = 7, 5.9%), a tumor involving both the anterior and middle fossae (*n* = 3, 2.5%), and the anterior fossa (*n* = 2, 1.7%).

Local invasion was documented in nearby structures as shown in 89 of 107 patients with available data (83.2%), particularly to the adjacent bone (*n* = 29, 32.6%), cavernous sinus (*n* = 26, 29.2%), and sphenoid sinus (*n* = 22, 24.7%). A histopathologic examination was available for all patients. All lesions were confirmed plasmacytoma, with inflammatory features noted in 25 (21%), lymphocytic infiltrate in 13 (11%), necrosis in 4 (3.4%), vasculitis in 3 (2.5%), and calcification in 3 (2.5%).

### 3.4. Management and Outcomes

Neoadjuvant therapy was not administered or not reported in 112 patients (94.9%). Chemotherapy was used as neoadjuvant therapy in 3 patients (2.5%), radiotherapy in 2 (1.7%), and combined chemoradiotherapy in 1 (0.8%).

The initial treatment consisted of surgery in 75 patients (63.6%), radiotherapy alone in 25 (21.2%), combined radiotherapy and chemotherapy in 15 (12.7%), and chemotherapy alone in 3 (2.5%). Those undergoing conventional radiotherapy received a median dose of 40 Gy (24–60 Gy), while data for stereotactic modality was limited. Among surgical cases (*n* = 75), subtotal resection (STR) was achieved in 35 (46.6%), gross total resection (GTR) in 27 (36%), and the extent of resection was not reported in 13 (17.3%). Intraoperative complications were rare, with no complications occurring in 72 cases (96%), hemorrhage in 2 (2.6%), and pituitary lesion in 1 (1.3%) (Table 4).

Adjuvant therapy was not specified or not used in 46 patients (38.9%). Conventional radiotherapy was used in 40 (33.8%), chemotherapy in 17 (14.4%), combined radiotherapy and chemotherapy in 14 (11.8%), and stereotactic radiosurgery in 1 (0.8%). Median dose among those receiving radiotherapy was 45 Gy (13–54 Gy), and fractioning was inconsistently reported. Post-treatment imaging was performed in 76 of 89 patients (85.4%), with 32 (42.1%) showing residual disease.

During follow-up, from the 89 patients who did not have a personal history of MM, the development of systemic disease occurred in 23 (19.5%), 55 (46.6%) had negative tests, and, for 11 (9.3%) patients, the source studies did not report progression or screening for MM. A single recurrence occurred in 22 (18.6%) patients and multiple recurrences in 1 (0.8%). The median time to last follow-up was 12 months (range, 0.35–120 months) with reports of having a resolution of symptoms in 45 patients (38.1%), an improvement in 31 (26.2%), worsening in 23 (19.5%), and symptom stability in 19 (16.1%). At the final status assessment (*n* = 118), 96 patients (81.4%) were alive and 22 (18.6%) had died.

### 3.5. Statistical Analysis

**Figure 3:**
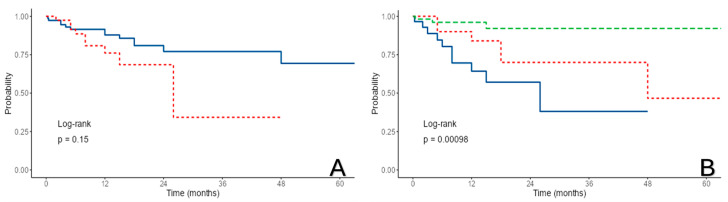
Survival probability for (**A**) initial treatment modality (blue = surgical, and red = non-surgical) and (**B**) multiple myeloma status (green = never developed multiple myeloma, red = developed after initial intervention, and blue = prior to plasmacytoma diagnosis).

On univariate analysis, associations between the status at last follow-up, recurrence status, and symptomatic control, with initial treatment modalities were assessed but no statistical differences were found (*p* values of 0.74, 0.4, and 0.66, respectively). Local invasion was not associated with the extent of resection (*p* = 0.13). Survival probability (Figure 3) was not statistically significant when comparing the initial treatment modality (surgical or non-surgical, *p* = 0.15), but a statistically significant difference (*p* < 0.01) was found when comparing the MM status.

Binary logistic regression identified MM history as the only statistically significant predictor of survival status at last follow-up. Patients who never developed MM had significantly higher odds of survival compared to those diagnosed with MM postoperatively (OR = 10.14, 95% CI: 1.63–63.04, *p* = 0.013). The tumor location, presence of local invasion, and initial treatment modality were not significantly associated with survival outcomes (Table 5).

## 4. Discussion

### 4.1. Patient Demographics and Epidemiological Insights

Na’ara et al. (2016) conducted a meta-analysis of 47 patients with skull base plasmacytomas, providing foundational insights into the demographics, clinical presentation, management strategies, and outcomes associated with this rare tumor [2]. Our expanded cohort of 118 patients builds upon this existing literature, offering a broader demographic and clinical spectrum. Rather than positioning this work as practice-changing, we frame its contribution as a descriptive, contemporary update to the existing literature and as a hypothesis-generating resource for a rare disease that cannot be studied prospectively at single centers. Relative to the foundational meta-analysis by Na’ara et al., the present synthesis adds value in several concrete respects. First, it expands the pooled sample size roughly two-and-a-half-fold (118 versus 47 patients), improving the precision with which clinical patterns can be described. Second, it documents a clear temporal shift in management toward operative intervention (surgery in 63.6% of our cohort versus 46.8% previously), reflecting the evolution of endoscopic and open skull base techniques. Third, it stratifies the multiple myeloma status by its timing relative to the skull base lesion (pre-existing versus subsequently developed, and preoperative versus postoperative diagnosis), allowing the prognostic weight of systemic disease to be examined more granularly than in prior work. Fourth, it expands the characterization of cranial-nerve involvement and patterns of local invasion, including structures and deficits not detailed in the earlier report. We acknowledge that these contributions are incremental and descriptive rather than definitive, and the associations reported here should be regarded as hypotheses to be tested in future prospective, multicenter collaborations.

Beyond describing clinical patterns in a larger cohort, this synthesis contributes a prognostic refinement. When the multiple myeloma status was stratified by its temporal relationship to the skull base lesion, patients who never developed myeloma had markedly higher odds of survival than those diagnosed with myeloma postoperatively (OR = 10.14, 95% CI: 1.63–63.04, *p* = 0.013), whereas a preoperative myeloma diagnosis conferred no significant survival difference relative to postoperative diagnosis (OR = 0.697, *p* = 0.622). This dissociation indicates that it is the presence of systemic disease, rather than its timing relative to local treatment, that governs prognosis, a distinction that reframes myeloma progression, rather than the local intervention chosen, as the dominant survival determinant and identifies myeloma conversion as the principal actionable endpoint for surveillance. Coupled with the documented shift toward operative management in the absence of a corresponding survival gain, these observations yield two clinically relevant implications for an ultra-rare disease: the escalation of surgical aggressiveness should be weighed against its unproven survival benefit, and structured long-term hematologic surveillance for myeloma conversion should be prioritized.

Both studies report a comparable median age of approximately 56 years; however, our cohort exhibits a slightly wider age range (19–81 years) compared to the 2016 cohort (28–85 years). While the previous study documented a male predominance of 59%, our analysis reveals a more balanced sex distribution, with males representing 55% of cases. These findings suggest a consistency in age-related epidemiology but indicate potential demographic shifts or subtle sampling variability in sex distribution [2].

### 4.2. Differential Pathologies

The diagnosis of skull base plasmacytoma remains a significant clinical challenge due to its substantial overlap in presentation and imaging characteristics with other skull base neoplasms, particularly chordomas and meningiomas [2]. Due to the local anatomy, these lesions commonly involve similar structures and present with similar symptoms, most commonly visual disturbances due to cranial nerve involvement [102,103]. However, due to differing pathology and disease progression, accurate diagnosis is critical for appropriate management, planning, and prognostic understanding. Chordomas are locally aggressive and have high recurrence rates [102]. Conversely, meningiomas are usually low-grade lesions with indolent growth and behavior [104].

In terms of patient demographics, skull base meningiomas have a female prevalence (2:1) while plasmacytomas and chordomas are more prevalent in males [102,105]. Other differentiating factors from imaging findings and immunohistochemistry (IHC) are instrumental in distinguishing plasmacytomas from these other neoplasms. On MRI imaging, plasmacytomas usually appear isointense on T1-weighted images and isointense to hyperintense on T2-weighted images, with a marked homogeneous contrast enhancement. Furthermore, clival destruction is frequently seen on computed tomography (CT) [106]. In comparison, chordomas are more likely to show a hypointense appearance on T1-weighted imaging with a heterogeneous enhancement [105]. Meningiomas appear isointense on both T1- and T2-weighted MRI, with cases commonly having an enhancing dural “tail” [105]. However, the presence of imaging findings mimicking a dural “tail” have been reported in cases of plasmacytoma, so this finding alone is not pathognomonic [106].

IHC findings often provide a key differentiating factor in the diagnoses of plasmacytomas of the skull base. They typically show light chain restriction and stain positive for CD138, CD38, and CD56. Notably, CD138 positivity identifies plasma cells, while CD56 is a marker that is usually negative in normal plasma cells [106]. Epithelial membrane antigen (EMA) may be positive in some cases of plasmacytomas, but this finding is more likely to be seen in skull base chordomas and meningiomas [107,108,109]. Chordomas additionally show high rates of cytokeratin and brachyury positivity on immunohistochemistry [110]. While an overlap in IHC findings is present, the presence of plasma cell markers is a specific feature that can confirm a diagnosis of plasmacytoma compared to other skull base lesions.

### 4.3. Tumor Location and Clinical Presentation

In both our cohort and the 2016 meta-analysis by Na’ara et al., visual disturbances emerged as the most common presenting symptom, reflecting the cranial nerve involvement associated with the lesion proximity to the optic pathway, cranial nerves, and related structures [2]. Although different location categorizations were used, the 2016 study reported that most of the included lesions were in the sphenoclival area (59.5%) or petrous apex (10.6%), which broadly aligns with our study, where most lesions were localized to the middle cranial fossa or posterior fossa. Anterior fossa involvement was rare in both studies, reported at 8.5% (4/47) in the 2016 report [2] and 4.2% (5/118) in our report.

In addition to visual symptoms, other neurologic deficits such as sensory and motor disturbances, along with hearing loss, were observed at lower rates. Abducens and oculomotor nerve involvement were prevalent in both reports, consistent with tumor encroachment on the parasellar and prepontine regions. Notably, our cohort exhibited higher rates of trigeminal (28.4%) and facial nerve (10.4%) involvement, with no included lesions impacting the olfactory nerve. In contrast, the 2016 report did not include any mention of trigeminal or facial nerve involvement but did have 10 cases (21.2%) of olfactory nerve involvement. Both studies reported cases of vestibulocochlear nerve involvement, however, at lower comparative rates, with symptoms manifesting as hearing loss, vertigo, and ataxia [2]. These patterns suggest a shared clinical phenotype centered on cranial neuropathies, while also highlighting potential anatomical and methodological variations that may influence the detection and reporting of specific nerve deficits.

### 4.4. Treatment Modalities and Clinical Outcomes

A notable divergence between our findings and those of Na’ara et al. (2016) lies in the evolving treatment landscape [2]. In the earlier cohort, the majority of patients (53%) received non-surgical management—radiotherapy alone (27.6%), chemotherapy alone (4.2%), or combined chemoradiotherapy (21.2%) [2]—with surgical resection performed in 47% of cases. In contrast, our study demonstrates a clear shift toward operative management, with 63.6% of patients undergoing surgical intervention. This trend likely reflects the advances in skull base surgical techniques and a growing inclination toward resection in selected patients.

Among those managed surgically, gross total resection (GTR) remained relatively infrequent in both cohorts, achieved in 36% of our cases and 45% in the 2016 study [2], underscoring the technical challenges posed by the proximity of these lesions to critical neurovascular structures. The high prevalence of local invasion observed in our cohort (83.2% of patients with available data) is a key contributor to this challenge. The most frequently invaded structures were adjacent bone (32.6%), the cavernous sinus (29.2%), and the sphenoid sinus (24.7%), which represent critical anatomical barriers to complete the resection at the skull base. Despite this, univariate analysis did not identify a statistically significant association between the presence of local invasion and extent of resection (*p* = 0.13), suggesting that other factors like tumor volume, approach selection, and surgeon experience may more directly determine the resectability in individual cases. Adjuvant radiotherapy remained a mainstay in both studies, regardless of the extent of the resection, reinforcing its role in multimodal treatment strategies.

Regarding the radiotherapy modality, most patients in our cohort who received radiation were treated with conventional fractionated radiotherapy, while only two patients received stereotactic radiosurgery as the initial modality and one as adjuvant therapy. Although emerging reports suggest that stereotactic modalities may offer effective local control with favorable toxicity profiles in selected skull base plasmacytoma cases [56,74,75], the predominance of conventional radiotherapy in our dataset precluded a meaningful comparative analysis between radiation modalities.

Despite the differences in the therapeutic approaches, the survival outcomes were broadly comparable. At a median follow-up of 12 months, 81.4% of patients in our cohort were alive, with 64.3% demonstrating symptomatic improvement or resolution. This is similar to findings from the prior report which reported a two-year survival of 78% and a five-year survival of 59% [2]. Furthermore, their multivariate analysis revealed no statistically significant association between surgical intervention or the extent of resection on the overall survival rate [2]. This finding correlates with the data seen in our report, with survival rates of our cohort being similar despite a higher proportion of patients treated with surgical resection.

Collectively, these observations suggest that, while surgical resection may offer symptomatic relief and local control in selected cases, its impact on long-term survival remains uncertain. This highlights the urgent need for prospective, ideally multicenter, studies to rigorously evaluate the comparative efficacy of surgical versus non-surgical modalities, and to define evidence-based guidelines for managing skull base plasmacytomas. Future research should also incorporate standardized functional outcome measures and long-term quality-of-life assessments, which are currently underreported. In addition, the integration of molecular profiling and imaging biomarkers may help stratify patients by recurrence risk and guide individualized therapeutic strategies.

A biological rationale helps explain why a higher proportion of surgical resection in our cohort did not translate into a measurable survival advantage. Plasmacytomas are composed of clonal plasma cells that are intrinsically radiosensitive, and definitive radiotherapy alone achieves durable local control in a large majority of solitary plasmacytomas, with most series reporting local control exceeding 80–90% at moderate doses on the order of 40–50 Gy [110]. Because radiotherapy reliably eradicates or controls the local lesion, cytoreductive surgery offers a limited incremental benefit for local control and has not been shown to improve overall survival; in this disease, long-term prognosis is instead driven chiefly by the progression to systemic multiple myeloma rather than by local tumor management [110,111,112,113].

Within this framework, the principal roles of surgery could be viewed as to establish a tissue diagnosis or, more importantly, to relieve the mass effect or neural compression. This paradigm could explain the comparable survival observed across treatment strategies in both our cohort and the earlier meta-analysis.

### 4.5. Multiple Myeloma History and Its Prognostic Significance

The relationship between MM status and survival emerged as the most clinically significant finding in this analysis. Among the 89 patients without a prior history of MM at presentation, 23 (19.5%) developed systemic disease after treatment, 55 (46.6%) remained disease-free at follow-up, and the MM screening status was not reported in 11 (9.3%). An additional 29 patients (24.6%) presented with a pre-existing MM diagnosis. Importantly, the timing of the MM diagnosis relative to the skull base plasmacytoma (preoperative or postoperative) carried distinct prognostic implications.

A Kaplan–Meier analysis demonstrated a statistically significant difference in overall survival when stratifying by MM status (*p* < 0.01). On multivariate binary logistic regression, MM history was the only independent predictor of survival status at last follow-up. Patients who never developed MM had 10-fold higher odds of survival compared to those with a postoperative MM diagnosis (OR = 10.14, 95% CI: 1.63–63.04, *p* = 0.013). In contrast, patients with a preoperative MM diagnosis did not demonstrate a statistically significant difference in survival compared to the postoperative MM group (OR = 0.697, *p* = 0.622), suggesting that, once systemic disease is established, its impact on prognosis may be independent of whether it preceded or followed treatment. These findings are consistent with the broader literature on solitary plasmacytoma, where MM progression is the primary determinant of long-term prognosis, with reported conversion rates of 50–60% within the first two years of diagnosis for solitary bone plasmacytoma [1,7]. The tumor location, presence of local invasion, and initial treatment modality were not independently associated with survival outcomes (Table 5), reinforcing that systemic disease burden, rather than local tumor characteristics, drives prognosis in this population. These findings underscore the critical importance of rigorous long-term surveillance for MM conversion in all patients treated for skull base plasmacytoma, and the need for a standardized prospective reporting of the timing of MM progression in future studies.

### 4.6. Limitations

This systematic review has several limitations. The included studies were predominantly case reports and small case series, which are susceptible to reporting, selection, and publication bias. The publication bias is likely substantial and directional: unusual presentations, favorable responses, and successfully managed cases are preferentially reported, so the pooled frequencies and survival estimates are probably biased toward optimism. The overall sample size, while the largest pooled cohort to date, limits the statistical power and contributes to the wide confidence intervals in the logistic regression model.

Outcome reporting was inconsistent across studies, limiting the interpretation of long-term results. Radiotherapy parameters were incompletely documented, as fractionation schemes were frequently omitted, precluding dose-response analysis. The near-exclusive use of conventional radiotherapy also precluded meaningful comparison of radiation modalities.

The timing of MM progression was inconsistently reported; authors frequently documented MM at follow-up without specifying the interval from treatment. All post-treatment MM cases were, therefore, grouped together, which may obscure the differences between early and late progressors. Functional outcomes were rarely reported using validated instruments, limiting the assessment of the treatment impact on recovery and quality of life.

Diagnostic criteria distinguishing solitary plasmacytoma, extramedullary plasmacytoma, and myeloma-associated disease were not uniformly applied across source studies, many of which predate current IMWG definitions. The pooled cohort is, therefore, biologically heterogeneous, and pooled estimates should be interpreted as describing a mixed plasma cell neoplasm population rather than a uniformly defined solitary plasmacytoma cohort.

Finally, the higher surgical proportion in our cohort alongside survival comparable to historical non-surgical series should not be interpreted as evidence of therapeutic equivalence or superiority. These are non-randomized retrospective data subject to confounding by indication, and no causal claim regarding any treatment modality is warranted.

## 5. Conclusions

This systematic review provides the largest pooled analysis to date of skull base plasmacytomas, elucidating their clinical presentation, anatomical distribution, treatment modalities, and outcomes. Our expanded cohort identified shifts in management, particularly increased surgical intervention. Despite this trend, survival outcomes remain comparable to prior data, a pattern that, while not establishing causation in these non-randomized data, is consistent with the limited additional survival benefit from resection alone. Stratifying myeloma status by its timing relative to the lesion further clarified that systemic disease burden, not the timing of its diagnosis or the local treatment chosen, is the dominant survival determinant, establishing myeloma conversion as the principal actionable endpoint for long-term surveillance. The rarity of these tumors and the predominance of low-level evidence underscore the need for prospective, multicenter studies with standardized reporting. Future research should prioritize molecular characterization and long-term functional outcomes to inform individualized, evidence-based management strategies.

## Figures and Tables

**Table 1:** Overview of included studies.

Study Author	Year	Study Design—Level of Evidence	Age	Sex	Duration of Symptoms, m	History of MM	Imaging Modality	Location of Tumor	Last FU, m	Symptoms at Last FU	Recurrence	Status
Sevindik et al. [31]	2024	Case report—V	64	M	No data	No	MRI	Posterior fossa	1	Improved	No	Alive
Ortega-Ruiz et al. [32]	2024	Case report—V	39	M	1	No	CT, MRI, PET	Middle fossa	4	Worsened	Yes	Alive
Shamas et al. [33]	2024	Case report—V	61	F	No data	No	CT, MRI	Posterior fossa	7	Resolved	No	Alive
DeJesus et al. [34]	2024	Case report—V	42	F	No data	Yes	MRI	Middle fossa	3	Worsened	No	Dead
Lesar et al. [35]	2024	Case report—V	21	F	3	No	CT, MRI, PET	Non- specified skull base	9	Resolved	No	Alive
Huang et al. [36]	2024	Case report—V	61	M	6	No	CT, MRI	Middle and posterior fossa	6	Improved	No	Alive
Chadli et al. [37]	2024	Case report—V	56	F	0.5	No	CT, MRI	Middle and posterior fossa	18	Resolved	Yes	Alive
Shah et al. [38]	2023	Case report—V	68	M	3	Yes	CT, MRI	Posterior fossa	0.75	Improved	No	Alive
Mansouri et al. [3]	2023	Case report—V	51	F	12	No	CT, MRI	Middle and posterior fossa	12	Resolved	No	Alive
Waqar et al. [39]	2022	Case report—V	60	M	0.11	Yes	CT, MRI	Posterior fossa	1	Resolved	No	Alive
Silverman et al. [40]	2022	Case report—V	60	M	0.5	No	CT, MRI	Middle fossa	5	Worsened	No	Dead
Khilji et al. [41]	2022	Case report—V	36	F	No data	No	CT, MRI, PET	Posterior fossa	3	Resolved	No	Alive
Sharifi et al. [42]	2022	Case report—V	62	F	3	No	CT, MRI	Middle fossa	6	Improved	No	Alive
Seetahal-Maraj et al. [43]	2022	Case report—V	56	M	0.25	No	CT, MRI	Middle fossa	3	Stable	Yes	Alive
Andaluz et al. [13]	2022	Case series—IV	59	M	11	No	CT, MRI	Posterior fossa	6	Resolved	No	Alive
			60	F	18	No	MRI	Posterior fossa	6	Worsened	Yes	Alive
Bonduelle et al. [44]	2021	Case report—V	67	M	0.18	No	CT, MRI	Middle fossa	3	Resolved	No	Alive
Kaufman et al. [45]	2021	Case report—V	44	M	0.75	Yes	MRI, PET	Middle fossa	7	Improved	No	Alive
Wang et al. [46]	2020	Case report—V	50	M	No data	No	CT, MRI	Posterior fossa	10	Improved	No	Alive
Jin et al. [14]	2019	Case Series—IV	50	M	0.66	No	CT, MRI	Posterior fossa	29	Improved	Yes	Alive
			59	F	1	No	CT, MRI	Posterior fossa	15	Resolved	No	Alive
			61	M	3	No	CT, MRI	Posterior fossa	120	Stable	Yes	Alive
			53	M	4	No	CT, MRI	Posterior fossa	14	Resolved	No	Alive
			47	F	5	No	CT, MRI	Posterior fossa	24	Resolved	No	Alive
Aoi et al. [47]	2019	Case report—V	65	F	6	No	CT, MRI	Middle fossa	18	Worsened	Yes	Dead
Pan et al. [48]	2019	Case report—V	76	F	No data	Yes	CT	Middle fossa	7	Improved	No	Alive
Ibekwe et al. [49]	2018	Case report—V	53	F	0.18	No	CT, MRI	Posterior fossa	3	Resolved	No	Alive
Siyag et al. [1]	2018	Case report—V	41	M	2	No	MRI, PET	Posterior fossa	12	Resolved	No	Alive
Cardoso et al. [12]	2018	Case series—IV	42	F	1	Yes	CT, MRI	Middle fossa	8	Worsened	Yes	Dead
			31	M	No data	Yes	MRI	Middle fossa	5	Worsened	Yes	Dead
Wu et al. [50]	2017	Case report—V	59	F	1	Yes	MRI	Middle fossa	18	Resolved	No	Alive
Amita et al. [51]	2017	Case report—V	55	M	3	No	MRI	Posterior fossa	8	Stable	No	Alive
Kumar et al. [52]	2017	Case report—V	50	F	3	No	CT, MRI	Middle fossa	18	Resolved	No	Alive
Lee et al. [15]	2017	Case Series—IV	62	M	1	No	MRI	Posterior fossa	3	Improved	No	Alive
			60	M	4	Yes	MRI	Middle fossa	2	Improved	No	Alive
			66	F	4	Yes	MRI	Middle fossa	6	Improved	No	Alive
			62	M	No data	Yes	MRI	Posterior fossa	36	Stable	No	Alive
			77	M	No data	Yes	MRI	Posterior fossa	12	Improved	No	Alive
Kadiri et al. [53]	2016	Case report—V	56	F	3	Yes	CT, MRI	Middle fossa	8	Worsened	No	Dead
Soejbjerg er al. [54]	2016	Case report—V	62	F	6	No	MRI, PET	Middle fossa	42	Stable	No	Alive
Kalwani et al. [55]	2015	Case report—V	69	M	0.25	No	CT, MRI, PET	Posterior fossa	18	Stable	Yes	Alive
Alafaci et al. [56]	2014	Case report—V	65	M	2	No	MRI	Posterior fossa	6	Resolved	No	Alive
Khalatbari et al. [57]	2014	Case report—V	46	M	0.5	No	CT, MRI	Middle fossa	48	Resolved	No	Alive
Jiang et al. [58]	2014	Case report—V	34	F	0.9	No	CT, MRI	Middle fossa	48	Resolved	No	Alive
Ozdemir et al. [59]	2013	Case report—V	50	M	1.5	No	CT, MRI	Middle fossa	12	Worsened	Yes	Dead
Ozturk et al. [16]	2013	Case series—IV	73	M	2	No	CT, MRI	Middle fossa	24	Resolved	No	Dead
			52	M	7	No	CT, MRI	Middle fossa	18	Resolved	No	Alive
Khan et al. [60]	2012	Case report—V	45	F	0.74	Yes	MRI	Middle fossa	14	Resolved	No	Alive
Guinto-Balanzar et al. [17]	2012	Case series—IV	66	F	3	No	MRI	Middle and posterior fossa	3	Worsened	No	Dead
			61	M	6	No	MRI	Posterior fossa	36	Improved	No	Alive
Liu et al. [61]	2012	Case report—V	40	M	No data	No	MRI	Posterior fossa	7	Improved	No	Alive
Kashyap et al. [18]	2010	Case series—IV	55	M	5	Yes	CT, MRI	Posterior fossa	6	Worsened	Yes	No data
			40	M	2	Yes	MRI	Posterior fossa	2	Improved	No	Alive
Liu et al. [62]	2010	Case report—V	54	F	1.4	No	MRI	Posterior fossa	22	stable	No	Alive
Fukai et al. [63]	2010	Case report—V	61	M	No data	No	CT, MRI	Middle fossa	96	Improved	No	Alive
Park et al. [64]	2009	Case report—V	32	M	0.5	No	CT, MRI, PET	Middle fossa	8	Resolved	No	Alive
Ko et al. [65]	2009	Case report—V	48	M	0.25	Yes	MRI	Middle fossa	15	Resolved	No	Dead
Rahmah et al. [66]	2009	Case report—V	33	M	12	Yes	XR, MR	Posterior fossa	12	Worsened	Yes	Dead
Terada et al. [67]	2009	Case report—V	81	F	No data	No	CT, XR, MRI, PET	Posterior fossa	18	Worsened	Yes	Dead
Zigouris et al. [68]	2009	Case report—V	78	F	6	No	CT, MRI	Anterior and Middle fossa	1	Resolved	No	Alive
Pitini et al. [69]	2008	Case report—V	65	M	2	No	MRI	Middle fossa	4	Stable	No	Alive
Yaman et al. [70]	2008	Case report—V	70	F	7	Yes	XR, MRI	Middle fossa	22	Resolved	No	Alive
Yamaguchi et al. [71]	2008	Case report—V	65	F	2	Yes	CT, XR, MRI	Middle and posterior fossa	17	Improved	No	Alive
Husein et al. [72]	2007	Case report—V	58	F	6	Yes	CT, MRI	Middle and posterior fossa	18	Resolved	No	Alive
Sinnott et al. [30]	2006	Case report—V	57	F	3	No	CT, MRI	Middle fossa	10	Stable	No	Alive
Richa Goyal et al. [73]	2006	Case report—V	60	M	2	No	CT	Posterior fossa	6	Resolved	No	Alive
Wong et al. [74]	2006	Case report—V	63	M	6	Yes	CT, MRI	Posterior fossa	12	Resolved	No	Alive
Duet et al. [19]	2005	Case series—IV	63	M	0.25	No	CT, MRI	Posterior fossa	12	Improved	No	Alive
Singh et al. [20]	2005	Case series—IV	38	F	8	Yes	MRI	Middle fossa	3	Improved	No	Alive
			42	M	No data	Yes	No data	Middle fossa	48	Resolved	No	Alive
Peker et al. [75]	2005	Case report—V	70	F	5	Yes	MRI	Middle fossa	26	Stable	No	Dead
Weilbaecher et al. [21]	2004	Case series—IV	63	M	No data	No	MRI	Middle fossa	5	Resolved	No	Dead
Higurashi et al. [22]	2004	Case series—IV	46	M	No data	No	CT, MRI, MRA	Middle and posterior fossa	26	Improved	No	Alive
			53	F	No data	No	CT, MRI, MRA	Middle and posterior fossa	30	Improved	No	Alive
Halefoglu et al. [76]	2004	Case report—V	54	M	No data	No	MRI	Middle fossa	1.5	No data	No	Alive
McLaughlin et al. [77]	2004	Case report—V	66	F	24	No	CT, XR	Middle fossa	108	Resolved	No	Alive
Hon et al. [78]	2003	Case report—V	45	F	0.25	No	MRI	Middle fossa	12	Resolved	No	Alive
Brannan et al. [23]	2003	Case series—IV	61	F	0.75	No	CT, MRI	Middle fossa	12	Stable	No	Alive
			50	M	2	No	MRI	Posterior fossa	8	Resolved	No	Alive
Ampil et al. [79]	2002	Case report—V	63	M	0.5	No	MRI	Middle fossa	10	Worsened	Yes	Alive
Hogan et al. [80]	2002	Case report—V	39	M	No data	Yes	MRI	Middle fossa	24	Resolved	No	Alive
Schwartz et al. [28]	2001	Case series—IV	43	F	No data	No	MRI	Middle fossa	31	Improved	No	Alive
			55	F	No data	No	MRI	Posterior fossa	28	Improved	No	Alive
			73	F	No data	No	MRI	Middle fossa	22	Improved	No	Alive
			49	M	No data	No	MRI	Posterior fossa	26	Worsened	Yes	Alive
			68	F	No data	No	MRI	Anterior fossa	4	Stable	No	Dead
			37	F	No data	No	MRI	Middle fossa	96	Resolved	No	Alive
Yetiser et al. [81]	2001	Case report—V	65	M	24	No	CT, MRI	Middle fossa	18	Resolved	No	Alive
Vijaya-Sekaran et al. [82]	1999	Case report—V	62	F	3	No	CT	Non-specified skull base	12	Improved	No	Alive
Mandagere et al. [83]	1998	Case report—V	53	F	3	No	CT	Middle fossa	84	Resolved	No	Alive
Chiang et al. [84]	1998	Case report—V	50	M	0.75	No	CT, MRI	Middle fossa	24	Stable	No	Alive
Okamoto et al. [24]	1997	Case series—IV	72	F	4	No	CT, MRI	Posterior fossa	48	Resolved	No	Alive
			64	M	1	No	CT, XR, MRI	Posterior fossa	8	Resolved	No	Alive
Mantyla et al. [85]	1996	Case report—V	67	F	0.75	Yes	CT	Non-specified skull base	0.35	Worsened	Yes	Dead
Bachmeyer et al. [86]	1996	Case report—V	77	M	36	Yes	CT, MRI	Middle fossa	6	Stable	No	Alive
Bindal et al. [29]	1995	Case series—IV	51	F	No data	No	No data	Middle fossa	96	Worsened	Yes	Alive
			30	M	No data	No	No data	Posterior fossa	3	Stable	Yes	Alive
			75	M	No data	No	No data	Posterior fossa	48	Worsened	No	Dead
Prasad et al. [25]	1994	Case series—IV	24	M	4	No	No data	Middle fossa	15	Worsened	No	Dead
			24	M	2	No	CT, XR	Anterior and middle fossa	14	Stable	No	Alive
Wax et al. [26]	1993	Case series—IV	65	M	0.75	No	No data	Non-specified skull base	19	Resolved	No	Alive
Brookes et al. [87]	1992	Case report—V	64	M	6	No	CT, XR	Non-specified skull base	18	Resolved	No	Alive
Losa et al. [88]	1992	Case report—V	50	F	0.75	No	CT, MRI	Middle fossa	18	Resolved	No	Alive
Du Preez et al. [27]	1991	Case series—IV	30	F	No data	No	CT, XR	Anterior and middle fossa	18	Resolved	No	Alive
			52	F	2	No	CT, XR	Anterior fossa	16	Resolved	No	Alive
Dhanani et al. [89]	1990	Case report—V	62	M	No data	No	CT, XR	Middle fossa	6	Worsened	No	Alive
Bellan et al. [90]	1989	Case report—V	71	M	0.75	Yes	CT	Middle fossa	2	Worsened	Yes	Dead
Bitterman et al. [91]	1986	Case report—V	42	M	No data	No	CT, MRI	Middle fossa	3	Improved	Yes	Alive
Evans et al. [92]	1985	Case report—V	66	M	0.75	No	CT, XR	Middle fossa	18	Improved	No	Alive
Humphrey et al. [93].	1983	Case report—V	60	M	No data	No	XR	Middle fossa	12	Worsened	Yes	Dead
Mancardi et al. [94]	1983	Case report—V	62	M	0.75	No	CT, XR, MRI	Middle and posterior fossa	18	Improved	No	Alive
Atweh et al. [95]	1982	Case report—V	30	F	No data	No	CT	Middle fossa	1	Improved	No	Alive
Vera et al. [96]	1980	Case report—V	49	M	0.1	No	CT, XR	Posterior fossa	14	Resolved	No	Alive
Toland et al. [97]	1971	Case report—V	19	M	6	No	XR	Non-specified skull base	24	Stable	No	Alive
Someren et al. [98]	1971	Case report—V	47	F	2	No	CT, XR, MRI	Posterior fossa	16	Stable	No	Alive
Weiner et al. [99]	1966	Case report—V	42	F	66	No	CT, XR, MRI	Middle fossa	12	Improved	No	Alive
Rovit et al. [100]	1960	Case report—V	55	F	60	No	CT, XR, MRI	Middle fossa	21	Improved	No	Alive
Cappell et al. [101]	1935	Case report—V	57	F	No data	No	No data	Non-specified skull base	0.5	Worsened	No	Dead

Abbreviations: M = male; F = female; m = month; CT = Computerized Tomography; FU = Follow-Up; GTR = Gross Total Resection; MM = Multiple Myeloma; MRA = Magnetic Resonance Angiography; MRI = Magnetic Resonance Imaging; PET = Positron Emission Tomography; STR = Subtotal Resection; XR = X-Rays.

**Table 2:** Summary of patient demographics and clinical symptoms.

Characteristics (No. of Patients for Whom Information Is Available)	Value (Among Patients with Available Data)
Cohort size (No.)	118
Demographics (*n* = 118)	
Median age, range (years)	56, 19–81
Gender (male), *n* (%)	65 (55)
Median duration of symptoms, range (*n* = 85)	2 months, 0.07–66 months
Personal History of Multiple Myeloma (*n* = 118)	No. (%)
Yes	29 (24.6)
No	89 (75.4)
Presenting Symptoms/Physical Exam Findings (*n* = 118)	No. (%) *
Visual Disturbances	78 (66.1)
Headache	64 (54.2)
Motor Disturbances	18 (15.3)
Palpable Mass/Swelling	15 (12.7)
Auditory Disturbance (tinnitus, hypoacusis)	13 (11)
Dizziness/Vertigo	13 (11)
Sensory Disturbance	10 (8.5)
Ataxia/Gait Disturbance	7 (5.9)
Mechanical Pain	8 (6.8)
Cognitive Disfunction	7 (5.9)
Dysphagia	6 (5.1)
Nausea/Vomiting	5 (4.2)
Nasal Discomfort (obstruction, epistaxis)	5 (4.2)
Speech Problem (dysarthria, aphasia)	3 (2.5)
Altered Deep Tendon Reflexes	2 (1.7)
Lymphadenopathy	1 (0.8)
Coughing	1 (0.8)
Tremor	1 (0.8)
Urinary Incontinence	1 (0.8)
Seizures	1 (0.8
Cranial Nerve Involvement (*n* = 67)	No. (%) *
Abducens	44 (65.7)
Trigeminal	19 (28.4)
Oculomotor	11 (16.4)
Facial	7 (10.4)
Optic	6 (9)
Trochlear	6 (9)
Vestibulocochlear	4 (6)
Hypoglossal	3 (4.5)
Vagus	1 (1.5)

* Some patients may have findings of multiple presenting symptoms and affected cranial nerves. Data represents the frequency of the individual findings in relation to the total sample size.

**Table 3:** Summary of clinical workup.

Characteristics (No. of Patients for Whom Information Is Available)	Value (Among Patients with Available Data)
Initial Imaging Modality (*n* = 113)	No. (%) *
MRI	92 (81.4)
T1W—hypointense	12 (10.6)
T1W—isointense	31 (27.4)
T1W—hyperintense	8 (7.0)
T2W—hypointense	6 (5.3)
T2W—isointense	15 (13.2)
T2W—hyperintense	16 (14.2)
CT	69 (61.1)
Xray	19 (16.8)
PET Scan	9 (7.9)
MRA	3 (2.6)
MRI based contrast enhancement (*n* = 118)	No. (%)
Homogeneous	50 (42.4)
Heterogeneous	17 (14.4)
No data	51 (43.2)
Location of Tumor (*n* = 118)	No. (%) *
Middle Fossa	57 (48.3)
Posterior Fossa	40 (33.9)
Middle and Posterior Fossa	9 (7.6)
Non-Specified (Skull Base)	7 (5.9)
Anterior & Middle Fossa	3 (2.5)
Anterior Fossa	2 (1.7)
Features of Local Invasion	No. (%)
Yes	89 (83.2) *
Adjacent bone	29 (32.6)
Cavernous sinus	26 (29.2)
Sphenoid sinus	22 (24.7)
Clivus	12 (13.5)
Meninges	10 (11.2)
Sellar region	10 (11.2)
Carotid artery	9 (10.1)
Optic apparatus	7 (7.9)
C1–C2 vertebrae	5 (5.6)
Auditory canal	4 (4.5)
Ethmoid sinus	4 (4.5)
Parasellar region	4 (4.5)
Temporal fossa	2 (2.2)
Adjacent soft tissues	2 (2.2)
Cerebellum	1 (1.1)
Cranial nerves	1 (1.1)
Jugular foramen	1 (1.1)
Frontal sinus	1 (1.1)
No	18 (15.1)
Histopathology Findings (*n* = 118)	No. (%) **
Neoplastic	117 (99.2)
Inflammatory Features	25 (21.2)
Lymphocytic Infiltrate	13 (11)
Necrosis	4 (3.4)
Vasculitis	3 (2.5)
Calcification	3 (2.5)

Abbreviations: CT = Computerized Tomography, MRA = Magnetic Resonance Angiography, MRI = Magnetic Resonance Imaging, PET = Positron Emission Tomography, T1W = T1-Protocol-weighted MRI, T2W = T2-Protocol-weighted MRI. * Some patients may have multiple imaging modalities and invasion of structures. Data represents the frequency of the individual findings in relation to the total sample size. ** Patients can have multiple histopathologic findings.

**Table 4:** Summary of management, complications, and outcomes.

Characteristics (No. of Patients for Whom Information Is Available)	Value (Among Patients with Available Data)
Neoadjuvant Therapy (*n* = 118)	No. (%)
No/Not Reported	112 (94.9)
Chemotherapy	3 (3.5)
Radiotherapy	2 (1.7)
Chemotherapy and Radiotherapy	1 (0.8)
Initial Treatment (*n* = 118)	No. (%)
Surgery	75 (63.6)
Radiotherapy	25 (21.2)
Radiotherapy + Chemotherapy	15 (12.7)
Chemotherapy	3 (2.5)
Median dose of radiotherapy as initial treatment, range (*n* = 33) *	40, 24–60 Gy
Extent of Resection (for surgical cases) (*n* = 75)	No. (%)
STR	35 (46.6)
GTR	27 (36)
Not Reported	13 (17.3)
Intraoperative Complications (*n* = 75)	No. (%)
None	72 (96)
Hemorrhage	2 (2.6)
Pituitary Lesion	1 (1.3)
Adjuvant Therapy (*n* = 118)	No. (%)
No/Not Specified	46 (38.9)
Conventional Radiotherapy	40 (33.8)
Chemotherapy	17 (14.4)
Radiotherapy + Chemotherapy	14 (11.8)
Stereotactic Radiosurgery	1 (0.8)
Median dose of adjuvant radiotherapy, range (*n* = 37) **	45, 13–54 Gy
MM progression after surgery (*n* = 118)	No. (%)
Yes	23 (19.5)
No	55 (46.6)
Unknown	11 (9.3)
Prior history	29 (24.6)
Imaging After Treatment (*n* = 89)	No. (%)
Yes	76 (85.4)
Residual Disease	32 (42.1)
No Residual Disease	34 (44.7)
Not Reported	10 (13.2)
No	13 (14.6)
Recurrent Disease (*n* = 118)	No. (%)
No	95 (80.5)
Single Recurrence	22 (18.6)
Multiple Recurrences	1 (0.8)
Median Time to Last Follow Up, range (*n* = 118)	12, 0.35–120 Months
Symptom Assessment at Last Follow Up (*n* = 118)	No. (%)
Resolved	45 (38.1)
Improved	31 (26.2)
Worsened	23 (19.5)
Stable	19 (16.1)
Status (*n* = 118)	No. (%)
Alive	96 (81.4)
Dead	22 (18.6

* Data not available for 9 patients. One patient received 21 Gy in 3 fractions of stereotactic radiosurgery while data for the other was missing. ** Data not available for 17 patients that received adjuvant radiation. One patient received stereotactic radiotherapy receiving 30 Gy in 2 fractions.

**Table 5:** Binary logistic regression analysis of predictors of survival in skull base plasmacytoma.

Predictor	OR	95% CI	*p*-Value
**Multiple Myeloma History** *(ref: postoperative diagnosis)*			
Preoperative diagnosis	0.697	0.166–2.92	0.622
Unknown	1.058	0.181–6.18	0.950
Never developed MM	10.139	1.631–63.04	0.013
**Tumor Location** *(ref: middle cranial fossa)*			
Posterior fossa	†	†	NE †
Middle & posterior fossa	†	†	NE †
Non-specified skull base	†	†	NE †
Anterior & middle fossa	†	†	NE †
Anterior fossa	†	†	NE †
**Local Invasion** *(ref: invasion present)*			
No local invasion	0.678	0.103–4.46	0.686
**Initial Treatment** *(ref: surgical resection)*			
Non-surgical	1.100	0.329–3.68	0.877

Note: Estimates represent the log odds of alive vs. deceased at last follow-up. † Tumor location estimates are unreliable due to complete separation. All deaths occurred within specific location subgroups, rendering odds ratios and confidence intervals non-estimable. These predictors were not statistically significant.

## Data Availability

All data generated or analyzed during this study are included in this article. Further inquiries may be directed to the corresponding author.

## Supplementary Materials

The following supporting information can be downloaded at: https://www.mdpi.com/article/10.3390/cancers18152495/s1, Supplementary File S1: Full Electronic Search Strategy. Supplementary File S2: PRISMA 2020 checklist. Reference [114] is cited in the Supplementary Materials.

## References

[1] Siyag A., Soni T.P., Gupta A.K., Sharma L.M., Jakhotia N., Sharma S. (2018). Plasmacytoma of the Skull-base: A Rare Tumor. Cureus.

[2] Na’ara S., Amit M., Gil Z., Billan S. (2016). Plasmacytoma of the Skull Base: A Meta-Analysis. J. Neurol. Surg. Part B Skull Base.

[3] Mansouri H., Elouaouch S., El Youssi Z., Guerrouaz M.A., Moukhlissi M., Berhili S., Mezouar L. (2023). Solitary plasmacytoma of the skull base: A case report and literature review. Radiol. Case Rep..

[4] Elsabah H., Ghasoub R., Soliman D.S., Ibrahim F., Aldapt M.B., Taha R.Y., Al Azawi S., Mudawi D., Moustafa A., Elomri H. (2024). Skull base plasmacytoma in young patients aged below 40 years: Radiological perspectives and clinical outcomes. Cancer Rep..

[5] Ma X.J., Li D., Wang L., Hao S.-Y., Zhang L.W., Zhang J.T., Wu Z. (2019). Clinical features, radiological profiles, and surgical outcomes of primary intracranial solitary plasmacytomas: A report of 17 cases and a pooled analysis of individual patient data. J. Neurooncol..

[6] Rajkumar S.V., Dimopoulos M.A., Palumbo A., Blade J., Merlini G., Mateos M.-V., Kumar S., Hillengass J., Kastritis E., Richardson P. (2014). International Myeloma Working Group updated criteria for the diagnosis of multiple myeloma. Lancet Oncol..

[7] Wein R.O., Popat S.R., Doerr T.D., Dutcher P.O. (2002). Plasma Cell Tumors of the Skull Base: Four Case Reports and Literature Review. Skull Base.

[8] Skerget S., Penaherrera D., Chari A., Jagannath S., Siegel D.S., Vij R., Orloff G., Jakubowiak A., Niesvizky R., Liles D. (2024). Comprehensive molecular profiling of multiple myeloma identifies refined copy number and expression subtypes. Nat. Genet..

[9] OCEBM Levels of Evidence Working Group (2011). The Oxford 2011 Levels of Evidence.

[10] Moola S., Munn Z., Tufanaru C., Aromataris E., Sears K., Sfetcu R., Currie M., Qureshi R., Mattis P., Lisy K., Aromataris E., Munn Z. (2017). Chapter 7: Systematic reviews of etiology and risk. Joanna Briggs Institute Reviewer’s Manual.

[11] Munn Z., Barker T., Moola S., Tufanaru C., Stern C., McArthur A., Stephenson M., Aromataris E. (2020). Methodological quality of case series studies: An introduction to the JBI critical appraisal tool. JBI Evid. Synth..

[12] Cardoso R.C., Gerngross P.J., Hofstede T.M., Weber D.M., Chambers M.S. (2014). The Multiple Oral Presentations of Multiple Myeloma. Support. Care Cancer.

[13] Andaluz L.D.M., Gonzalez J.A.C., Ramírez Z.E.S., Ramírez N., Castellanos L.G., Estrada E.M.E. (2022). Solitary bone plasmacytoma as posterior fossa cranial neoplasia, presentation of two clinical cases. Surg. Neurol. Int..

[14] Jin L., Gui S., Li C., Bai J., Cao L., Liu C., Wang X., Zhang Y. (2019). Differential Diagnosis and Treatment Modality of Parasellar Plasmacytoma: Clinical Series and Literature Review. World Neurosurg..

[15] Lee J., Kulubya E., Pressman B.D., Mamelak A., Bannykh S., Zada G., Cooper O. (2017). Sellar and clival plasmacytomas: Case series of 5 patients with systematic review of 65 published cases. Pituitary.

[16] Öztürk K., Şahin M., Midilli R., Gürsan G., Özsan N., Savaş R. (2013). Extramedullary Plasmacytoma of Head and Neck Region: Report of Six Cases with Different Localizations. Int. J. Otorhinolaryngol. Clin..

[17] Guinto-Balanzar G., Abdo-Toro M., Aréchiga-Ramos N., Leal-Ortega R., Zepeda-Fernández E., Nambo-Lucio M.d.J. (2012). Plasma cell tumor of the clivus: Report of two cases. Cir. Cir..

[18] Kashyap R., Kumar R., Kumar S. (2010). Cranial nerve palsy in multiple myeloma and solitary plasmacytoma. Asia-Pac. J. Clin. Oncol..

[19] Duet M., Tran Ba Huy P., Wassef M., Liote F. (2005). Plasma Cell Problems. J. Clin. Oncol..

[20] Singh A.D., Chacko A.G., Chacko G., Rajshekhar V. (2005). Plasma cell tumors of the skull. Surg. Neurol..

[21] Weilbaecher C., Patwardhan R.V., Fowler M., Willis B.K., Nanda A. (2004). Metastatic lesions involving the sella: Report of three cases and review of the literature. Neurol. India.

[22] Higurashi M., Yagishita S., Fujitsu K., Kitsuta Y., Takemoto Y., Osano S. (2004). Plasma cell myeloma of the skull base: Report of two cases. Brain Tumor Pathol..

[23] Brannan S.O., Matthews B.N., Savant V., Brown R.D., Matthews T.D. (2003). Solitary Intracranial Extra-osseous Plasmacytoma Presenting with Ophthalmic Signs. J. Neuroophthalmol..

[24] Okamoto K., Ito J., Furusawa T., Sakai K., Tokiguchi S., Sato M., Tanaka R., Nemoto K., Oyanagi K. (1997). Solitary plasmacytomas of the occipital bone: A report of two cases. Eur. Radiol..

[25] Prasad M.L., Mahapatra A.K., Kumar L., Khosla A., Sarkar C., Prasad A., Roy S. (1994). Solitary intracranial plasmacytoma of the skull base. Indian. J. Cancer.

[26] Wax M.K., Yun K.J., Omar R.A. (1993). Extramedullary plasmacytomas of the head and neck. Otolaryngol. Neck Surg..

[27] Du Preez J.H., Branca E.P. (1991). Plasmacytoma of the Skull: Case Reports. Neurosurgery.

[28] Schwartz T.H., Rhiew R., Isaacson S.R., Orazi A., Bruce J.N. (2001). Association between Intracranial Plasmacytoma and Multiple Myeloma: Clinicopathological Outcome Study. Neurosurgery.

[29] Bindal A.K., Bindal R.K., van Loveren H., Sawaya R. (1995). Management of intracranial plasmacytoma. J. Neurosurg..

[30] Sinnott B.P., Hatipoglu B., Sarne D.H. (2006). Intrasellar plasmacytoma presenting as a non-functional invasive pituitary macro-adenoma: Case report & literature review. Pituitary.

[31] Sevindik B., Uysal E., Ugras S., Sevindik B., Uysal E., Ugras S. (2024). A Rare Tumor Causing Optic and Oculomotor Nerve Compression: Clivus Plasmacytoma Case Report. Cureus.

[32] Ortega-Ruiz O.R., Olivas J.A.L., Sangrador-Deitos M.V., Magaña R.M., Gurria J.A.R., Amador J.L.G. (2024). Combined endoscopic transorbital and transnasal approach for the management of a solitary plasmacytoma of the sphenoid bone: A case report and literature review. Surg. Neurol. Int..

[33] Shamas T., Reynolds C.D., Garcia R.R., Doescher R., Fuchs D., Rogers S.N. (2024). Markedly T2-Hypointense Clival Plasmacytoma With Light Chain Deposition Disease: A Case Report. Cureus.

[34] De Jesus O. (2024). Multiple myeloma extramedullary relapse at the sellar and suprasellar region after autologous stem cell transplantation. Surg. Neurol. Int..

[35] Lesar U., Janse van Rensburg L., Oelofsen S., McCree K., Ackerman C., Davis R. (2024). Multiple myeloma in a young female presenting as an aggressive skull-base tumour. S. Afr. J. Radiol..

[36] Huang T.R., Lin C.S., Chen H.C. (2024). Solitary Bone Plasmacytoma of the Skull Base With an Unusual Presentation. Ear Nose Throat J..

[37] Chadli S., Oudrhiri M.Y., Maamar M., Boutarbouch M., Khibri H., Haidouri S., Messaoud O., El-Aoufir O., Melhaoui A., Ammouri W. (2024). Sphenoid plasmacytoma as initial presentation of multiple myeloma—Case report. J. Surg. Case Rep..

[38] Shah C.P., Chamlagain R., Shah S., Paudel S., Sah S.K., Koirala B., Pandit K., Sitaula S., Shrestha A. (2023). Multiple myeloma with plasmacytoma of the clivus bone presenting with multiple cranial nerve III, IV, and VI palsy: A diagnostic dilemma. Clin. Case Rep..

[39] Bin Waqar S.H., Rehan A., Salahi N., Zhonghua L., McFarlane I. (2022). An Exceptional Case of Diplopia and Ptosis: Extramedullary Plasmacytoma of the Clivus With Multiple Myeloma. Cureus.

[40] Silverman R.F., Hanson L., Salahi N., Li Z., Boruk M., Hodgson N.M. (2022). Case Report: Cavernous Sinus Syndrome as the Initial Presentation of Multiple Myeloma. Front. Ophthalmol..

[41] Khilji H., Silver C., Morrar D., Chhabra A.M., Mandel S., Langer D.J., Shani D., Ellis J.A. (2022). Complete Resolution of Skull Base Solitary Plasmacytoma Using Proton-Beam Radiotherapy: A Case Report. Cureus.

[42] Sharifi S., Asadi M. (2022). Extramedullary plasmacytoma of the sphenoid sinus presenting with visual loss: A case report. Clin. Case Rep..

[43] Seetahal-Maraj P., Knight P., Ramnarine N. (2022). Proptosis secondary to a solitary plasmacytoma of the sphenoid bone: A case report on a rare skull base tumour. Egypt. J. Neurosurg..

[44] Bonduelle Q., Garas G., Ramakrishnan Y. (2021). A rare cause of diplopia: Solitary extramedullary plasmacytoma of the skull base. Ann. R. Coll. Surg. Engl..

[45] Kaufman A.R., Quillen K., Distefano A.G., Sloan J.M. (2021). Nonsecretory Recurrence of Multiple Myeloma Presenting as Sixth Nerve Palsy Secondary to Clival Plasmacytoma. J. Neuroophthalmol..

[46] Wang L., Ren N.J., Cai H., Cheng H.F., Zhang H.L., Peng X.B., He Z.W. (2020). Solitary plasmacytoma of the occipital bone: A case report. J. Int. Med. Res..

[47] Aoi I. (2018). Solitary Skull Base Plasmacytoma with Sella Turcica Extension. Int. J. Gerontol..

[48] Pan W.Y., Wang T.Y., Tsai C.C. (2019). Sphenoidal Plasmacytoma Associated with Multiple Myeloma Mimicking Meningeal Tumor in a Geriatric Patient. Int. J. Gerontol..

[49] Ibekwe E., Horsley N.B., Jiang L., Achenjang N.-S., Anudu A., Akhtar Z., Chornenka K.G., Monohan G.P., Chornenkyy Y.G. (2018). Abducens Nerve Palsy as Initial Presentation of Multiple Myeloma and Intracranial Plasmacytoma. J. Clin. Med..

[50] Wu P.W., Lee T.J., Chen J.R., Huang C.C. (2017). An unusual presentation of multiple myeloma with unilateral sudden vision loss: A case report. Medicine.

[51] Amita R., Sandhyamani S., Nair S., Kapilamoorthy T.R. (2017). Plasmacytoma of the clivus. Asian J. Neurosurg..

[52] Kumar R., Kumar N., Mohindro S., Radotra B.D. (2017). Solitary plasmacytoma of temporal bone: A rare case report. Asian J. Neurosurg..

[53] Kadiri S. (2016). Multiple Myeloma Revealed by a Sphenoid Plasmocytoma: Case Report and Literature Review. Integr. J. Med. Sci..

[54] Soejbjerg A., Dyve S., Baerentzen S., Thorsell G., Poulsen P.L., Jorgensen J.O.L., Kampmann U. (2016). The solitary sellar plasmacytoma: A diagnostic challenge. Endocrinol. Diabetes Metab. Case Rep..

[55] Kalwani N., Remenschneider A.K., Faquin W., Ferry J., Holbrook E.H. (2015). Plasmacytoma of the Clivus Presenting as Bilateral Sixth Nerve Palsy. J. Neurol. Surg. Rep..

[56] Alafaci C., Grasso G., Conti A., Caffo M., Salpietro F.M., Tomasello F. (2014). Cyberknife radiosurgery for cranial plasma cell tumor. Turk. Neurosurg..

[57] Khalatbari M.R., Hamidi M., Moharamzad Y. (2014). Gradenigo’s syndrome as first presentation of solitary osseous plasmacytoma of the petrous apex. Arch. Iran. Med..

[58] Jiang C.Z., Lin Q.S., Wu X.Y., Wang C.Y., Kang D.Z. (2014). Sellar Solitary Plasmacytoma Progressing to Multiple Myeloma: A Case Report and Literature Review. Medicine.

[59] Özdemir S., Tarkan Ö., Tuncer Ü., Sürmelioğlu Ö., Doğrusöz M., Ergin M. (2013). A case of extramedullary plasmacytoma in the sphenoid sinus with unilateral loss of vision. J. Cranio-Maxillofac. Surg..

[60] Khan I.S., Javalkar V., Thakur J.D., Nanda A. (2012). Intrasellar plasmacytoma: An illustrative case and literature review. J. Clin. Neurosci..

[61] Liu Y., Qiu J. (2012). Solitary Intracranial Plasmacytoma Located in the Clivus: A Diagnostic and Theraputic Challenge. N. Am. J. Med. Sci..

[62] Liu Z., Qi X., Wu X., Luo C., Lu Y. (2010). Solitary Intracranial Plasmacytoma Located in the Spheno-Clival Region Mimicking Chordoma: A Case Report. J. Int. Med. Res..

[63] Fukai J., Nohgawa M., Uematsu Y., Itakura T., Kamei I. (2010). Immunoglobulin D Multiple Myeloma Involving the Sella Manifesting as Oculomotor Palsy: Case Report. Neurosurgery.

[64] Park S.H., Kim Y.Z., Lee E.H., Kim K.H. (2009). Endoscopic Endonasal Transsphenoidal Resection of Solitary Extramedullary Plasmacytoma in the Sphenoid Sinus with Destruction of Skull Base. J. Korean Neurosurg. Soc..

[65] Ko S., Huang S.Y., Liu C.Y. (2009). Extramedullary plasmacytoma masquerading as Tolosa–Hunt syndrome: A case report. Case Rep..

[66] Rahmah N., Brotoarianto H., Andor E., Kusnarto G., Muttaqin Z., Hongo K. (2009). Dural plasmacytoma mimicking meningioma in a young patient with multiple myeloma. Biomed. Imaging Interv. J..

[67] Terada T. (2009). Multiple myeloma presenting as an intracranial plasmacytoma: A case report. Cases J..

[68] Zigouris A., Drosos D., Alexiou G.A., Fotakopoulos G., Mihos E., Pahatouridis D., Tsiouris S., Fotopoulos A.D., Voulgaris S. (2009). Primary plasmacytoma of the cranial vault: A case report. Cases J..

[69] Pitini V., Arrigo C., Alafaci C., Altavilla G. (2008). Extramedullary plasmacytoma presented as a non-functional invasive pituitary macro-adenoma. J. Neurooncol..

[70] Yaman E., Benekli M., Coskun U., Sezer K., Ozturk B., Kaya A.O., Yildiz R., Uluoglu O., Buyukberber S. (2008). Intrasellar plasmacytoma: An unusual presentation of multiple myeloma. Acta Neurochir..

[71] Yamaguchi S., Terasaka S., Ando S., Shinohara T., Iwasaki Y. (2008). Neoadjuvant therapy in a patient with clival plasmacytoma associated with multiple myeloma: A case report. Surg. Neurol..

[72] Husein O.F., Jacob A., Massick D.D., Welling D.B. (2007). Recurrence of Isolated Multiple Myeloma in the Skull Base: A Case Report and Review of the Literature. Ear Nose Throat J..

[73] Goyal R., Gupta R., Radotra B.D. (2006). Plasmacytoma of the clivus: A case report. Indian J. Pathol. Microbiol..

[74] Wong E.T., Lu X.Q., Devulapalli J., Mahadevan A. (2006). Cyberknife Radiosurgery for Basal Skull Plasmacytoma. J. Neuroimaging.

[75] Peker S., Abacıoğlu U., Bayraklı F., Kılıç T., Pamir M.N. (2005). Gamma knife radiosurgery for cavernous sinus plasmacytoma in a patient with breast cancer history. Surg. Neurol..

[76] Halefoglu A.M. (2004). Solitary extramedullary plasmacytoma arising in the pterygoid fossa. Acta Otolaryngol..

[77] McLaughlin D.M., Gray W.J., Jones F.G.C., Mirakhur M., McCance D.R., Sheridan B., Atkinson A.B. (2004). Plasmacytoma: An unusual cause of a pituitary mass lesion. A case report and a review of the literature. Pituitary.

[78] Hon C., Au W.Y., Shek T.W. (2003). Isolated sphenoid plasmacytoma presenting as painful ophthalmoplegia. Haematologica.

[79] Ampil F.L., Borski T.G., Nathan C.A.O., Mulcahy G., Walker M., Chin H.W., Stucker F.J. (2002). Cavernous Sinus Involvement by Extramedullary Plasmacytoma of the Sphenoid Sinus. Leuk. Lymphoma.

[80] Hogan M.C., Lee A., Solberg L.A., Thomé S.D. (2002). Unusual presentation of multiple myeloma with unilateral visual loss and numb chin syndrome in a young adult. Am. J. Hematol..

[81] Yetiser S., Talas D., Akkaya A., Deveci S. (2001). Plasmacytoma of the Temporal Bone: Management with a Combination of Surgery and Radiotherapy. Acta Otolaryngol..

[82] Vijaya-Sekaran S., Delap T., Abramovich S. (1999). Solitary plasmacytoma of the skull base presenting with unilateral sensorineural hearing loss. J. Laryngol. Otol..

[83] Mandagere K.A., Schimke R.N., Kyner J.L., Bhatia P.S. (1998). An Unusual Sellar Mass—Solitary Plasmacytoma. Endocr. Pract..

[84] Chiang S.K., Canalis R.F., Ishiyama A., Eversole L.R., Becker D.P. (1998). Plasmacytoma of the temporal bone. Am. J. Otolaryngol..

[85] Mäntylä R., Kinnunen J., Böhling T. (1996). Intracranial plasmacytoma: A case report. Neuroradiology.

[86] Bachmeyer C., Levy V., Carteret M., Laccourreye O., Danel C., Tourneau A.L., Zittoun R., Grateau G. (1997). Sphenoid sinus localization of multiple myeloma revealing evolution from benign gammopathy. Head. Neck.

[87] Brookes G.B., Marais J., Lee C.-C. (1992). Solitary Plasmacytoma of the Skull Base. Ann. Otol. Rhinol. Laryngol..

[88] Losa M., Terreni M.R., Tresoldi M., Marcatti M., Campi A., Triulzi F., Scotti G., Giovanelli M. (1992). Solitary plasmacytoma of the sphenoid sinus involving the pituitary fossa: A case report and review of the literature. Surg. Neurol..

[89] Dhanani A.-N.N., Bilbao J.M., Kovacs K. (1990). Multiple myeloma presenting as a sellar plasmacytoma and mimicking a pituitary tumor: Report of a case and a review of the literature. Endocr. Pathol..

[90] Bellan L.D., Cox T.A., Gascoyne R.D. (1989). Parasellar syndrome caused by plasma cell leukemia. Can. J. Ophthalmol..

[91] Bitterman P., Ariza A., Black R.A., Allen W.E., Lee S.H. (1986). Multiple myeloma mimicking pituitary adenoma. Comput. Radiol..

[92] Evans P.J., Jones M.K., Hall R., Scanlon M.F. (1985). Pituitary function with a solitary intrasellar plasmacytoma. Postgrad. Med. J..

[93] Humphrey D.M., Aufdemorte T.B., Gates G.A. (1983). An igd extramedullary plasmacytoma involving the sphenoid sinus at onset: An immunohistochemical study. Laryngoscope.

[94] Mancardi G.L., Mandybur T.I. (1983). Solitary intracranial plasmacytoma. Cancer.

[95] Atweh G.F., Jabbour N. (1982). Intracranial Solitary Extraskeletal Plasmacytoma Resembling Meningioma. Arch. Neurol..

[96] Vera C.L., Kempe L.G., Powers J.M. (1980). Plasmacytoma of the clivus presenting with an unusual combination of symptoms. J. Neurosurg..

[97] Toland J., Phelps P.D. (1971). Plasmacytoma of the skull base. Clin. Radiol..

[98] Someren A., Osgood C.P., Brylski J. (1971). Solitary posterior fossa plasmacytoma: Case report. J. Neurosurg..

[99] Weiner L.P., Anderson P.N., Allen J.C. (1966). Cerebral plasmacytoma with myeloma protein in the cerebrospinal fluid. Neurology.

[100] Rovit R.L., Fager C.A. (1960). Solitary Plasmacytoma of Petrous Bone. J. Neurosurg..

[101] Cappell D.F., Mathers R.P. (1935). Plasmacytoma of the Petrous Temporal Bone and Base of Skull. J. Laryngol. Otol..

[102] Zhao L., Chang C., Zhuang Y., Wang B., Qin L., Zheng J.-J., You Y.-P., Liu N., Ji J., Zheng K. (2023). Primary Skull Base Chordomas: A Clinicopathological Analysis of 94 Patients. World Neurosurg..

[103] Ilyas A., Przybylowski C., Chen C.J., Ding D., Foreman P.M., Buell T.J., Taylor D.G., Kalani M.Y., Park M.S. (2019). Preoperative embolization of skull base meningiomas: A systematic review. J. Clin. Neurosci..

[104] Meling T.R., Da Broi M., Scheie D., Helseth E. (2019). Meningiomas: Skull base versus non-skull base. Neurosurg. Rev..

[105] Thust S.C., Yousry T. (2016). Imaging of skull base tumours. Rep. Pract. Oncol. Radiother..

[106] Gagliardi F., Losa M., Boari N., Spina A., Reni M., Terreni M.R., Mortini P. (2013). Solitary clival plasmocytomas: Misleading clinical and radiological features of a rare pathology with a specific biological behaviour. Acta Neurochir..

[107] Ouyang T., Zhang N., Zhang Y., Jiao J., Ren J., Huang T., Chen J. (2014). Clinical Characteristics, Immunohistochemistry, and Outcomes of 77 Patients with Skull Base Chordomas. World Neurosurg..

[108] Ülgen E., Bektaşoğlu P.K., Sav M.A., Can Ö., Danyeli A.E., Hızal D.B., Pamir M.N., Özduman K. (2019). Meningiomas Display a Specific Immunoexpression Pattern in a Rostrocaudal Gradient: An Analysis of 366 Patients. World Neurosurg..

[109] Zuo Z., Tang Y., Bi C.F., Zhang W.Y., Zhao S., Wang X.Q., Yang Q.P., Zou L.Q., Liu W.-P. (2011). Extraosseous (extramedullary) plasmacytomas: A clinicopathologic and immunophenotypic study of 32 Chinese cases. Diagn. Pathol..

[110] Wang K., Tian K., Wang L., Wu Z., Ren C., Hao S., Feng J., Li J., Wan H., Jia G. (2015). Brachyury: A sensitive marker, but not a prognostic factor, for skull base chordomas. Mol. Med. Rep..

[111] Soutar R., Lucraft H., Jackson G., Reece A., Bird J., Low E., Samson D. (2004). Guidelines on the diagnosis and management of solitary plasmacytoma of bone and solitary extramedullary plasmacytoma. Br. J. Haematol..

[112] Ozsahin M., Tsang R.W., Poortmans P., Belkacémi Y., Bolla M., Dinçbas F.Ö., Landmann C., Castelain B., Buijsen J., Curschmann J. (2006). Outcomes and patterns of failure in solitary plasmacytoma: A multicenter Rare Cancer Network study of 258 patients. Int. J. Radiat. Oncol. Biol. Phys..

[113] Elsayad K., Oertel M., König L., Hüske S., Le Ray E., Meheissen M.A., Elsaid A.A., Elfaham E., Debus J., Kirova Y. (2020). Maximizing the clinical benefit of radiotherapy in solitary plasmacytoma: An international multicenter analysis. Cancers.

[114] Page M.J., McKenzie J.E., Bossuyt P.M., Boutron I., Hoffmann T.C., Mulrow C.D., Shamseer L., Tetzlaff J.M., Akl E.A., Brennan S.E. (2021). The PRISMA 2020 statement: An updated guideline for reporting systematic reviews. BMJ.

