# Supplementary material for: Skull-Base Plasmacytomas: A Systematic Review on Therapeutic Trends
Source: Cancers (Basel). 2026 Aug 4;18(15):2495. doi: 10.3390/cancers18152495 (PMC13465630; doi:10.3390/cancers18152495)
Supplement: Supplementary file 1 [file cancers-18-02495-s001.zip › Supplementary File S1.pdf]

Supplementary File S1

Full Electronic Search Strategy

**Initial Boolean term:** ("plasmacytoma" OR "extramedullary plasmacytoma" OR "solitary bone plasmacytoma") AND ("skull base" OR "clivus" OR "sphenoid" OR "petrous apex")

| Concept | Controlled vocabulary | Free-text (title/abstract) terms |
|---|---|---|
| **A. Plasmacytoma** | PubMed MeSH: "Plasmacytoma"[Mesh] EMBASE Emtree: 'plasmacytoma'/exp | plasmacytoma; extramedullary plasmacytoma; solitary bone plasmacytoma; solitary plasmacytoma |
| **B. Skull base** | PubMed MeSH: "Skull Base"[Mesh] EMBASE Emtree: 'skull base'/exp | skull base; cranial base; clivus; sphenoid; sphenoid sinus; petrous apex; clival |

*Concepts A and B were combined with AND. Within each concept, synonyms were combined with OR.*

**PubMed / MEDLINE**

#1 "Plasmacytoma"[Mesh]
#2 "plasmacytoma"[tiab] OR "extramedullary plasmacytoma"[tiab] OR "solitary bone plasmacytoma"[tiab] OR "solitary plasmacytoma"[tiab]
#3 #1 OR #2
#4 "Skull Base"[Mesh]
#5 "skull base"[tiab] OR "cranial base"[tiab] OR "clivus"[tiab] OR "clival"[tiab] OR "sphenoid"[tiab] OR "petrous apex"[tiab]
#6 #4 OR #5
#7 #3 AND #6

**Single-line equivalent**

("Plasmacytoma"[Mesh] OR plasmacytoma[tiab] OR "extramedullary plasmacytoma"[tiab] OR "solitary bone plasmacytoma"[tiab]) AND ("Skull Base"[Mesh] OR "skull base"[tiab] OR clivus[tiab] OR sphenoid[tiab] OR "petrous apex"[tiab])

**EMBASE**
#1 'plasmacytoma'/exp
#2 (plasmacytoma OR 'extramedullary plasmacytoma' OR 'solitary bone plasmacytoma' OR 'solitary plasmacytoma').tw.
#3 #1 OR #2
#4 'skull base'/exp
#5 ('skull base' OR 'cranial base' OR clivus OR clival OR sphenoid OR 'petrous apex').mp.
#6 #4 OR #5
#7 #3 AND #6

**Web of Science Core Collection**
TS = ("plasmacytoma" OR "extramedullary plasmacytoma" OR "solitary bone plasmacytoma" OR "solitary plasmacytoma")
AND
TS = ("skull base" OR "cranial base" OR "clivus" OR "sphenoid" OR "petrous apex")

**Cochrane**
#1 (plasmacytoma OR "extramedullary plasmacytoma" OR "solitary bone plasmacytoma"):ti,ab,kw
#2 ("skull base" OR clivus OR sphenoid OR "petrous apex"):ti,ab,kw
#3 #1 AND #2
